# Universal vs Targeted Chlorhexidine Bathing and Nasal Decolonization in Hospitalized Patients

**DOI:** 10.1001/jamanetworkopen.2025.0341

**Published:** 2025-03-10

**Authors:** Lyndon P. James, Natasha K. Stout, Taliser R. Avery, Sarah Stein, Kenneth E. Sands, Edward J. Septimus, Julia Moody, Eunice J. Blanchard, Russell E. Poland, Richard Platt, Susan S. Huang

**Affiliations:** 1 Boston University Chobanian & Avedisian School of Medicine, Boston, Massachusetts; 2 Center for Health Decision Science, Harvard T. H. Chan School of Public Health, Boston, Massachusetts; 3 Department of Population Medicine, Harvard Pilgrim Health Care Institute, Boston, Massachusetts; 4 Harvard Medical School, Boston, Massachusetts; 5 HCA Healthcare, Nashville, Tennessee; 6 Texas A&M College of Medicine and Memorial Hermann Health System, Houston, Texas; 7 Division of Infectious Diseases, University of California Irvine School of Medicine, Irvine

## Abstract

**Importance:**

The ABATE Infection trial investigated the effects of universal bacterial decolonization with chlorhexidine for patients in non–intensive care unit settings to reduce hospital-onset bacteremia and fungemia (HOB) events. Among patients with medical devices (central venous catheters, midline catheters, and lumbar drains), universal decolonization (UD) resulted in a significant and meaningful reduction in bacteremia compared with the standard of care (SOC), but cost-effectiveness is unclear.

**Objective:**

To examine the cost-effectiveness of universal and targeted bathing strategies compared with SOC in general medical and surgical units.

**Design, Setting, and Participants:**

A decision analytic model was constructed from June 1, 2021, to May 31, 2024, to simulate the frequency of HOB and costs under 3 strategies: SOC, UD, and targeted decolonization (TD). The model included a simulated cohort representative of the cluster-randomized ABATE Infection trial, which involved more than 500 000 participants across the US.

**Main Outcomes and Measures:**

In TD, decolonization was administered for patients with medical devices only. Upstream costs of bathing and downstream costs of HOB, under payer and hospital perspectives were included. Parameters were informed by the ABATE Infection Trial and additional literature. Willingness-to-pay per HOB prevented was adopted as $25 000 for payers and $10 000 for hospitals. Sensitivity analyses were tailored to populations with different characteristics.

**Results:**

The simulated cohort, based on the population from the ABATE trial, included 529 000 adult admissions with a mean (SD) age of 63 (18) years, 54% female, and 13% with a central venous catheter, midline catheter, or lumbar drain. In the base case, the SOC was least effective and most costly. Targeted decolonization was least costly and UD resulted in the fewest HOB events. Targeted decolonization was the cost-effective strategy from payer and hospital perspectives. Compared with TD, UD had an incremental cost-effectiveness ratio of $119 700 per HOB averted from the payer perspective, and $126 600 per HOB averted from the hospital perspective. Depending on willingness-to-pay, UD may be preferred in scenarios with a higher proportion of patients with medical devices, greater reductions in HOB from decolonizing in those with devices, and lower adherence under TD.

**Conclusions and Relevance:**

In this decision analytic model studying universal and targeted bathing, TD was cost-effective under a broad range of scenarios for both hospital system and payer decision-makers. Universal decolonization was cost-effective in some scenarios, such as in specific units where many patients have medical devices or if it were difficult to implement a targeted approach.

## Key Points

**Question:**

What is the cost-effectiveness of universal and targeted chlorhexidine bathing and nasal decolonization to prevent hospital-onset bacteremia and fungemia (HOB) in general medical and surgical units?

**Findings:**

In a decision analytical model including a simulated cohort, a targeted strategy using chlorhexidine only for patients with medical devices was cost-effective in most cases, while universal bathing was cost-effective in settings of high device prevalence, higher willingness-to-pay to prevent HOB, and lower adherence to targeted bathing. The standard of care was seldom cost-effective.

**Meaning:**

The findings of this study suggest that, in most general inpatient units, chlorhexidine bathing and nasal decolonization may be a cost-effective way to reduce HOB, particularly when targeted to patients with medical devices.

## Teaser

This decision analytical model was constructed to examine the cost-effectiveness of universal and targeted chlorhexidine bathing and nasal decolonization in hospitalized patients not being cared for in the intensive care unit.

## Introduction

Hospital-onset bacteremia and fungemia (HOB) are a major source of mortality, morbidity, and health care costs in the US.^1^ Use of decolonization products, most commonly chlorhexidine gluconate antiseptic bathing soap to remove body surface bacteria plus mupirocin ointments to remove nose bacteria, have been shown to reduce HOB and other infections. The REDUCE MRSA (methicillin-resistant *Staphylococcus aureus*) cluster-randomized pragmatic clinical trial found that universal decolonization (UD) of patients in intensive care units (ICUs) was highly effective at preventing bloodstream infections compared with either screening and isolation alone or screening and isolation followed by targeted decolonization (TD) of MRSA carriers.^2^ A subsequent cost-effectiveness analysis found that the practice was not only cost-effective but also cost-saving (ie, more effective at reducing infections and less costly) for hospitals in the US.^3^

More recently, the ABATE Infection trial examined the same practice of UD for medical and surgical inpatients outside the ICU compared with the standard of care (SOC).^4^ The cluster randomized trial involved more than 500 000 patients (340 000 patients in the intervention period alone, across 53 US community hospitals in 14 states), concluding there was no significant effect of UD on bacteremia outcomes for the trial population. However, in a post hoc subgroup analysis, decolonization was associated with a reduction in bacteremia in patients with central venous catheters, midline catheters, and lumbar drains (hereinafter referred to as medical devices). Alongside these results, financial penalties levied by the Centers for Medicare & Medicaid Services based on the quality metric of central-line–associated bloodstream infection may also have contributed to the widespread implementation of decolonization in the US, where 63% of hospitals had adopted the practice in some form as of 2021.^5^ Some national guidance now recommends targeted bathing for patients with devices in non-ICU settings.^6^

Decision modeling can explore important tradeoffs between universal and targeted approaches. A universal approach (ie, the trial intervention) establishes a uniform standard with an economy of scale that could reduce costs and may also prevent other infections.^7,8,9^ Conversely, a targeted approach focuses resources to those most likely to benefit, and this may overcome the lower adherence often associated with targeted approaches. We conducted a decision analysis to inform policymakers and hospital decision-makers about the relative benefits, costs, and cost-effectiveness of applying universal and targeted decolonization strategies in adult medical and surgical units compared with status quo bathing.

## Methods

We constructed a decision tree to estimate HOB infections and costs in non-ICU settings under 3 strategies: UD, TD, and SOC (eTable 1 in Supplement 1). Model parameters were obtained from the ABATE Infection trial and additional sources (Table 1^4,10,11,12,13,14,15,16^; eTable 2 in Supplement 1). Because all data were obtained from published literature, this study did not constitute human research and does not require institutional review board review or exemption according to the Common Rule (45 CFR §46). We followed the Consolidated Health Economic Evaluation Reporting Standards (CHEERS) reporting guideline for health economic evaluation.^17^ The present study was conducted from June 1, 2021, to May 31, 2024.

**Table 1.** Model Input Parameters

Variable description	Point estimate^a^	Distribution for probabilistic sensitivity analysis	Source
**HOB infection**			
Probability of HOB		β	Secondary analysis of ABATE Infection trial baseline period data
With device			
MRSA-positive, surgical	0.110	Events, No. = 18; nonevents, No. = 146	
MRSA-positive, nonsurgical	0.081	Events, No. = 73; nonevents, No. = 831	
MRSA-negative, surgical	0.015	Events, No. = 100; nonevents, No. = 6759	
MRSA-negative, nonsurgical	0.023	Events, No. = 338; nonevents, No. = 14 371	
Without device			
MRSA-positive, surgical	0.023	Events, No. = 5; nonevents, No. = 214	
MRSA-positive, nonsurgical	0.019	Events, No. = 47; nonevents, No. = 2416	
MRSA-negative, surgical	0.002	Events, No. = 54; nonevents, No. = 34 283	
MRSA-negative, nonsurgical	0.002	Events, No. = 258; nonevents, No. = 129 168	
HRR for HOB, those with device		Log-normal mean (SD) of log HRR	Secondary analysis of ABATE Infection trial
Treated	0.86	0.153 (0.059)	
Untreated	1.14	0.129 (0.066)	
Probability of medical device	0.125	Fixed	Secondary analysis of ABATE Infection trial
Probability of history of MRSA	0.080	Fixed	Livorsi et al,^10^ 2021
Probability of surgery	0.217	Fixed	ABATE Infection trial,^4^ intervention period
Proportion of surgical patients receiving CHG bathing as standard of care	0.5	Fixed	Assumption, preoperative bathing performed
Adherence to universal decolonization			
CHG	78%	Fixed	ABATE Infection trial^4^
Mupirocin	88%		
Adherence to targeted decolonization			
Relative to universal decolonization (CHG and mupirocin)	90%	Fixed	Assumption
Absolute			
CHG	71%		
Mupirocin	79%		
**Costs (2022 US$ unless otherwise specified)** ^11^			
Excess cost of HOB, payer perspective	$19 643 (2006 US$^b^)	Normal 95% CI, 9026-30 260	Kilgore et al,^12^ 2008
Excess cost of HOB, hospital perspective	$25 000	NA	Assumption; tested in sensitivity analyses
Willingness-to-pay to prevent a single HOB event, payer perspective	$25 000	NA	Assumption; tested in sensitivity analyses
Willingness-to-pay to prevent a single HOB event, hospital perspective	$10 000	NA	Assumption; tested in sensitivity analyses
Cost of CHG bed bath, prepackaged, per day	$5.52	γ	Petlin et al,^13^ 2014
		Mean (SD), 5.52 (1.84)	
Cost of CHG, 4% shower, 118-mL bottle to last 2 d	$0.875	γ	Petlin et al,^13^ 2014
		Mean (SD), 0.875, SD 0.292	
Cost of standard bed bath, prepackaged, per day	$2.11	γ	Larson et al,^14^ 2004
		Mean (SD), 2.11 (0.70)	
Cost of shower soap, per day	$0.44	γ	Larson et al,^14^ 2004
		Mean (SD), 0.44 (0.147)	Petlin et al,^13^ 2014
Proportion of bed baths vs showers	78%	Fixed	ABATE Infection Trial^4^
Cost of twice-daily intranasal mupirocin for 5 d	$6.23	γ	Courville et al,^15^ 2012
		Mean (SD), 6.23 (2.08)	Young et al,^16^ 2006
Hospital length of stay	6.5 d	Log normal	ABATE Infection trial^4^
		Mean (SD), 6.5 (0.724) to match IQR, 4-8 d	

Abbreviations: CHG, chlorhexidine gluconate; HOB, hospital-onset bacteremia and fungemia; HRR, hazard rate ratio; MRSA, methicillin-resistant *Staphylococcus aureus*; NA, not applicable.

^a^ Point estimate indicates the mean of 10 000 sets of results.

^b^ Updated to 2022 US$ in model.

### Population

In the base case, we modeled the ABATE Infection trial population, consisting of adult admissions to US non-ICU units over 12-month baseline and 21-month intervention periods in 2013-2016 (Table 1), where 12.5% of patients had a medical device and 21.7% were admitted for a surgical reason. We assumed 8% had current or historical evidence of colonization with MRSA in keeping with published norms.^18,19^ The baseline probability of HOB associated with various combinations of these 3 characteristics was obtained from secondary analysis of the ABATE Infection trial data (Taliser Avery, MS; Harvard Pilgrim Health Care Institute; private communication; July 31, 2021). In sensitivity analyses, we varied the proportion of patients with these characteristics.

### Bathing Strategies

We compared 3 bathing strategies. Standard of care represented the status quo bathing practices in the routine care arm of the trial, comprising nonantiseptic disposable soap cloths for bed baths and nonantiseptic liquid soap for showering. Under the SOC, surgical patients received chlorhexidine bathing at 50% adherence during their total inpatient stay (accounting for preoperative but not postoperative bathing). No mupirocin administration was assigned to the SOC group.

As per the ABATE Infection trial,^4^ the UD strategy exchanged routine soap for rinse-off chlorhexidine, 4%, in showers and leave-on chlorhexidine, 2%, disposable cloths for bed bathing for all patients. All those with a history of MRSA also received twice-daily nasal mupirocin, 2%, ointment for 5 days. In the TD strategy, the chlorhexidine bathing described for the UD strategy was applied only to patients with medical devices, and mupirocin was given only to patients with devices who had a history of MRSA. All other patients were bathed according to SOC (eTable 1 in Supplement 1).

### Cost and Health-Related Outcomes

Costs and HOB infections were reported per 1000 admissions. As in the ABATE Infection trial, HOB events were included for sample collection dates more than 2 days after unit admission through 2 days after unit discharge. We conducted the analysis using a health care payer perspective, including all costs resulting from the consumption of health care goods and services, regardless of whether these are borne by the provider or insurer.^20,21^ In scenario analyses, we examined cost impacts from a hospital perspective, focusing on costs likely to be borne by hospitals.^20^

Total costs included upstream costs for bathing and nasal decolonization as well as downstream costs associated with HOB. Upstream costs were modeled in the same way under payer and hospital perspectives. Downstream costs were informed by a published analysis for the payer perspective,^12^ which provided an estimate for all HOB events (rather than other estimates limited to central-line–associated bloodstream infection) along with a 95% CI for uncertainty analysis. For the hospital perspective, we assumed that the cost accruing to the hospital for each HOB event was $25 000, including all nonreimbursed care costs and financial penalties.^22^ All costs were inflated to 2022 US dollars using the Consumer Price Index for medical care.^23^ As a secondary outcome, we reported the number of individuals treated with mupirocin per 1000 admissions.

### Cost-Effectiveness Analysis

We determined the cost-effective strategy according to convention.^20,24^ First, we ranked by increasing cost. Next, any so-called dominated strategies were ruled out (ie, those with both more HOB events and higher cost than another strategy). For all remaining strategies, incremental cost-effectiveness ratios were calculated as the additional cost of a strategy divided by the number of HOB events prevented, relative to the next cheapest, nondominated strategy. We then identified the cost-effective strategy as that with the highest incremental cost-effectiveness ratio underneath the willingness-to-pay threshold. We assumed that payers had a willingness-to-pay threshold of $25 000 per HOB averted, and hospitals had a willingness-to-pay threshold of $10 000 per HOB event prevented.

### Key Model Assumptions

Conditional on device status, we assumed the effect of decolonization on HOB was the same for UD and TD; that is, the same benefit was provided to those with devices who receive the intervention, regardless of whether those without devices on the unit receive decolonization. No other positive health effects of decolonization were incorporated beyond HOB. In addition, no harmful health effects were assigned to decolonization, due to the very low proportion of patients with adverse events reported in both the ABATE Infection trial (25 mild events among 183 013 patients receiving chlorhexidine) and the REDUCE MRSA trial (7 mild events among 29 000 patients receiving chlorhexidine).^2,4^

Adherence in the UD strategy was set to the adherence reported in the ABATE Infection trial, which was designed as a pragmatic trial where implementation was performed by usual hospital staff. Universal decolonization adherence was 79% for chlorhexidine bathing and 88% for mupirocin administration, and TD adherence was assumed to be worse than UD. Bathing-related workload would not be expected to differ between UD and the SOC, but under TD there is an additional time cost due to the extra workflow step of identifying specified patients and ensuring they have the correct type of bathing equipment to deliver a targeted strategy. In the base case, TD adherence was set to 90% of adherence relative to that achieved under UD (ie, absolute adherence, 71% for chlorhexidine bathing). Imperfect adherence was assumed to be distributed randomly across the population.

### Tailoring to Different Contexts

We varied key parameters to test how cost-effectiveness differed beyond the base case assumptions, depending on the patient population served or other factors relevant for decision-making. In one-way deterministic sensitivity analyses, we varied each of the following key parameters, holding others constant: proportion of patients with medical devices who had a history of MRSA colonization and were admitted to a surgical ward, adherence to bathing under TD compared with UD, and willingness-to-pay to prevent each HOB event.

We also performed a 4-way deterministic sensitivity analysis, varying the following parameters at the same time within plausible ranges for US hospitals: proportion of patients with medical devices (range, 12.5%-50%), reduction in hazard rate ratio of HOB among those with devices who received the decolonization intervention (range of hazard rate ratio reduction, 0%-50%), willingness-to-pay to prevent a single HOB (range, $0-50 000), and adherence to decolonization under TD compared with UD (range, 40%-71% absolute or 50%-90% compared with UD).

### Statistical Analysis

In the base case, we accounted for uncertainty across model input parameters using probabilistic sensitivity analysis.^25^ We encoded the uncertainty for each model input parameter with a distribution informed by the literature wherever possible and meaningful (Table 1; eTable 2 in Supplement 1). We sampled 10 000 sets of values from these distributions and ran the model once for each set. We then obtained our point estimates as the mean of 10 000 sets of results and constructed 95% uncertainty intervals (UIs) as 2.5th and 97.5th centiles. Analyses were performed using TreeAge Pro 2023 Healthcare Version (TreeAge Software LLC)^26^ and R, version 4.3.3 (R Foundation for Statistical Computing).^27,28,29,30,31,32,33,34,35,36^

## Results

The characteristics of the modeled population have been published.^4^ There were 529 000 total admissions with a mean (SD) age of 63 (18) years, and 54% were female.

### Base Case

There were 6.6 HOB events per 1000 admissions under the SOC (95% UI, 6.1-7.2 per 1000 admissions). Universal decolonization resulted in an HOB rate of 5.8 per 1000 admissions (95% UI, 5.3-6.3 per 1000 admissions). Under TD, the estimated HOB rate was 5.9 per 1000 admissions (95% UI, 5.4- 6.4 per 1000 admissions) (Table 2). Targeted decolonization was the least costly strategy under both analytic perspectives. From the payer perspective, the cost per 1000 admissions was $232 100 under the SOC (95% UI, $112 900-$356 300 per 1000 admissions), $219 900 per 1000 admissions under UD (95% CI, $109 100-$351 300 per 1000 admissions), and $210 000 under TD (95% UI, $102 800-$325 000 per 1000 admissions). From the hospital perspective, the cost per 1000 admissions was $186 600 under the SOC (95% UI, $161 700-$239 200 per 1000 admissions), $180 000 per 1000 admissions under UD (95% CI, $145 900-$266 300 per 1000 admissions), and $169 600 under TD (95% UI, $144 300-$224 800 per 1000 admissions) (Table 2). In eTable 3 in Supplement 1, costs are presented separately for the categories of intervention-related (ie, upstream) and HOB-related (ie, downstream) costs.

**Table 2.** Cost-Effectiveness of Universal Decolonization, Targeted Decolonization, and Standard of Care for HOB Prevention^a^

Strategy^b^	HOB Events		Perspective, point estimate (95% UI)			
			Payer		Hospital	
	Per 1000 admissions (95% UI)	Averted	Total cost per 1000 admission, $	ICER, $ per HOB prevented	Total cost per 1000 admissions, $	ICER, $ per HOB prevented
TD	5.9 (5.4-6.4)	Baseline comparator	210 000 (102 800-325 000)	Baseline comparator	169 600 (144 300-224 800)	Baseline comparator
UD	5.8 (5.3-6.3)	0.082 (0.076-0.090)	219 900 (109 100-351 300)	119 700	180 000 (145 900-266 300)	126 600
SOC	6.6 (6.1-7.2)	Results in more HOB	232 100 (112 900-356 300)	More costly and results in more HOB	186 600 (161 700-239 200)	More costly and results in more HOB

Abbreviations: HOB, hospital-onset bacteremia and fungemia; ICER, incremental cost-effectiveness ratio; SOC, standard of care; TD, targeted decolonization; UD, universal decolonization; UI, uncertainty interval.

^a^ Strategies listed in order of increasing cost, in 2022 US dollars. Total costs included upstream costs for bathing and nasal decolonization as well as downstream costs associated with each HOB. Definitions of upstream and downstream costs appear in the Methods section.

^b^ Strategies are described in the eMethods section and eTable 1 in Supplement 1.

From the payer’s perspective, the SOC was dominated by UD and TD (ie, both bathing strategies usually cost less than SOC while also averting more HOB events). Compared with TD, UD was expected to avert an additional 0.082 HOB events (95% UI, 0.076-0.090 events) at an additional cost of $9900 per 1000 admissions (95% UI, −$4900 to $49 700 per 1000 admissions) from the payer perspective, reflecting an average incremental cost-effectiveness ratio of $119 700 per HOB event averted. From the hospital perspective, UD was estimated to cost an additional $10 400 compared with TD per 1000 admissions (95% UI, −$4200 to $49 900 per 1000 admissions), giving an incremental cost-effectiveness ratio of $126 600 per HOB event averted (Table 2). The number of individuals receiving mupirocin under the SOC was 0, compared with 7.9 per 1000 admissions under TD and 70.4 per 1000 admissions under UD.

### Tailored Analyses

**Figure 1.**
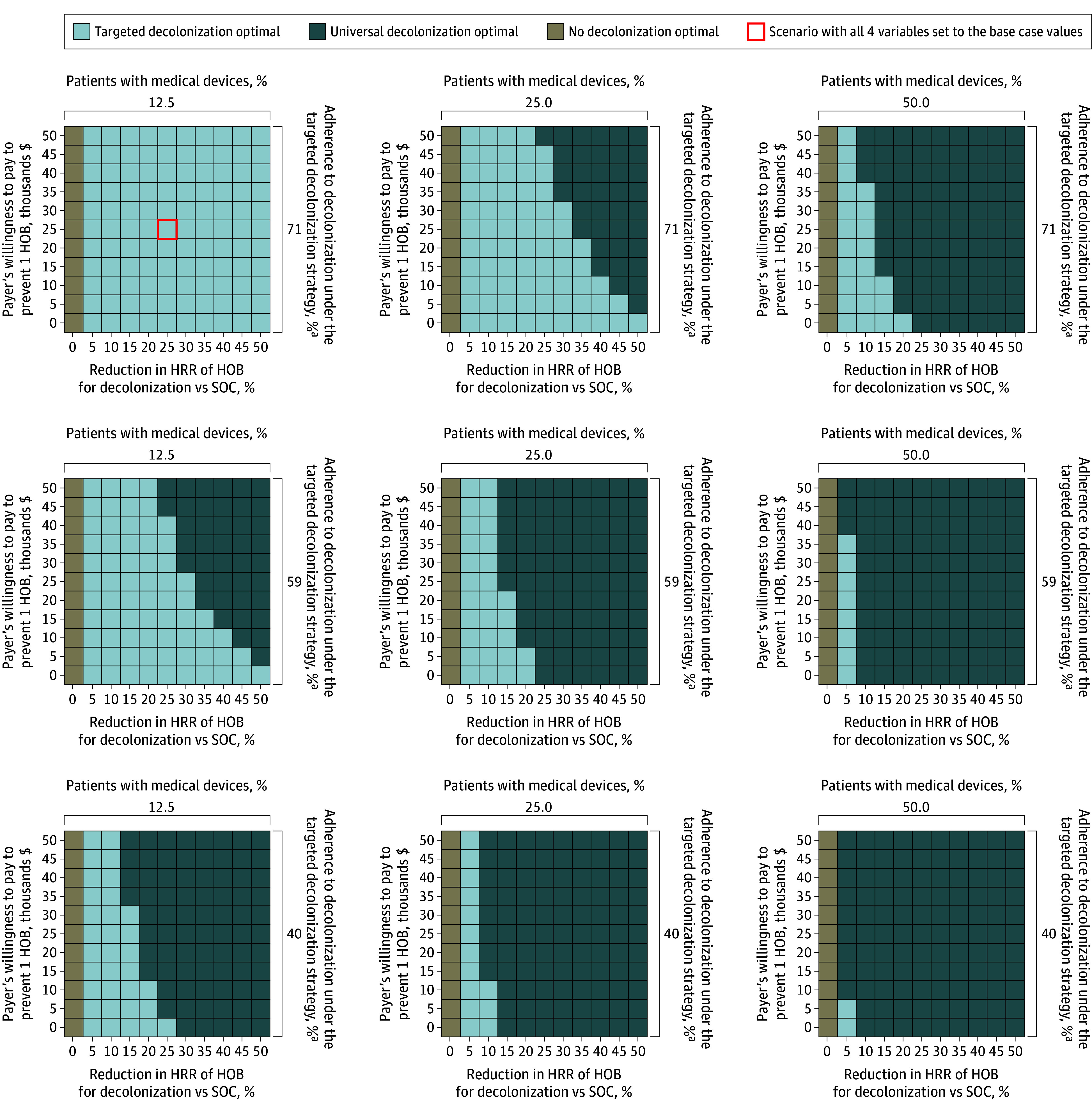
Key Parameters and Optimal Bathing Strategy, Payer Perspective The optimal strategy (ie, that which provided the best value for money) is shown for the payer perspective as a function of the proportion of patients with devices, adherence to decolonization under the targeted decolonization strategy, the treatment effect for decolonization among those with devices, and the payer’s willingness-to-pay to avoid 1 episode of hospital-onset bacteremia and fungemia (HOB). All other variables are held at base case values. HRR indicates hazard rate ratio; SOC, standard of care. ^a^Adherence values of 40%, 59%, and 71% refer to absolute adherence and equate to 50%, 75%, and 90% relative to 79% adherence to chlorhexidine bathing under universal decolonization experienced in the ABATE Infection Trial.

Figure 1 and the eFigure in Supplement 1 show the variation of the optimum strategy across 4 key model parameters from both payer and hospital perspectives. As the proportion of non-ICU patients with devices increased and as the success of targeting these patients with devices for decolonization decreased, UD was found to be the cost-effective strategy. In addition, as the hospital’s willingness-to-pay threshold to prevent HOB increased, and as the intervention became more effective in preventing HOB than estimated in the trial, the cost-effectiveness of the UD strategy exceeded that of TD. Overall, from the payer perspective, UD was favored in approximately two-thirds of the explored parameter scenarios, and UD was favored in half the parameter scenarios explored from the hospital perspective.

**Figure 2.**
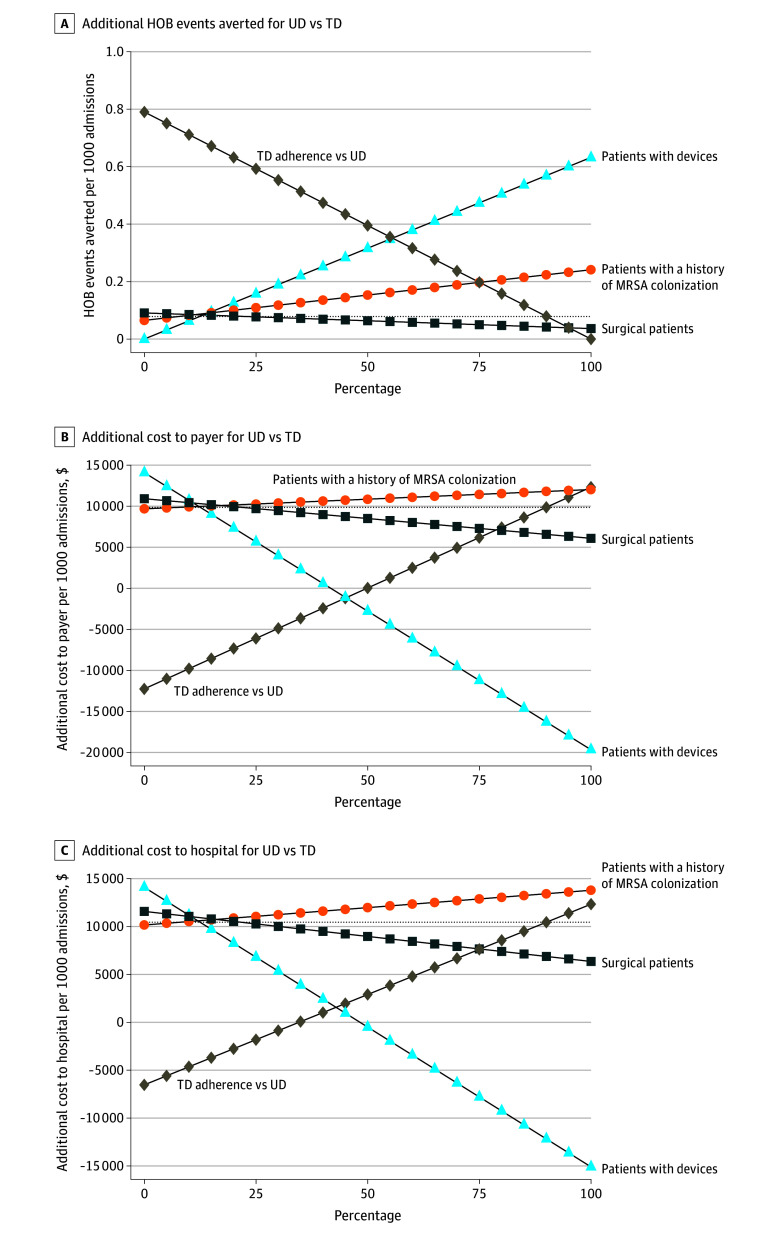
Single Parameters and the Incremental Benefits and Costs of Universal Decolonization (UD) Compared With Targeted Decolonization (TD) Incremental hospital-onset bacteremia and fungemia (HOB) events prevented (A), incremental costs from the payer perspective (B), and incremental costs from the hospital perspective (C) are shown across key variable ranges. Adherence under TD is plotted compared with adherence under UD. For each deterministic sensitivity analysis plotted, all other variables are held at base case values. Base case results indicated by dotted lines.

For hospital and payer decision-makers considering the size of potential benefits and costs of UD compared with TD across different patient populations, the results of key one-way sensitivity analyses on the incremental number of HOB events averted and the incremental costs of UD compared with TD are shown in Figure 2. HOB events averted and costs averted under the UD strategy increased most substantially with increasing device prevalence and decreasing adherence to TD compared with UD. The proportion of patients undergoing surgery or having a history of MRSA had opposing and more modest effect estimates.

**Figure 3.**
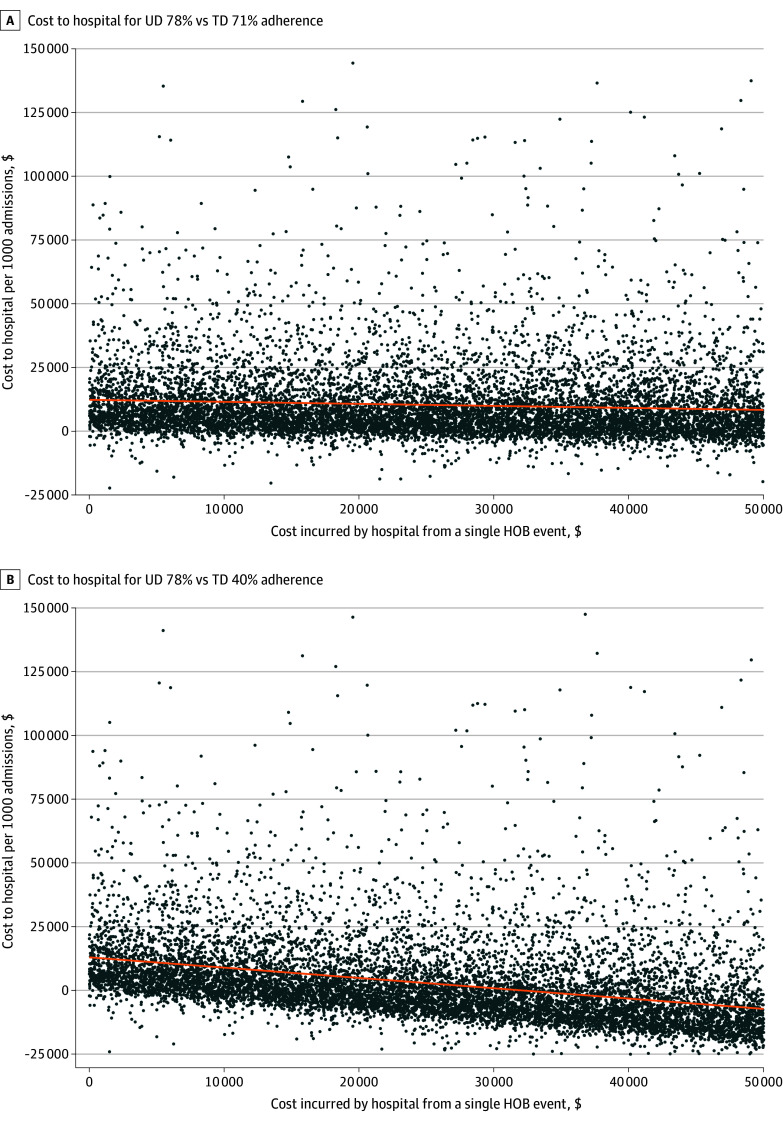
Incremental Cost of Universal Bathing Per 1000 Admissions, Compared With Targeted Bathing, From the Hospital Perspective A, Targeted decolonization (TD) 71% adherence vs universal decolonization (UD) 78% adherence. B, TD 40% vs UD 78% adherence. Each blue point represents 1 of 10 000 probabilistic sensitivity analysis iterations per plot. Orange lines are constructed as a simple, least-squares regression of the y-variable on the x-variable. The x-axis in each subplot is the average net cost to the hospital for a hospital-onset bacteremia and fungemia (HOB) event, incorporating all amounts reimbursed and all HOB-related penalties.

Figure 3 shows how the incremental cost for UD compared with TD was affected by the net cost accrued to the hospital per HOB event from the hospital perspective. As the net hospital cost of an HOB increased, this did not greatly impact the incremental cost of UD when adherence under TD was high. However, at a lower level of adherence, UD resulted in lower overall cost than TD when the net cost incurred by the hospital was $35 000 or higher.

## Discussion

In this decision analytic model studying the cost-effectiveness of universal and targeted chlorhexidine bathing and nasal decolonization in non-ICU settings, we modeled a population based on more than 500 000 patients in the 53-community hospital ABATE Infection trial. Overall, a decolonization protocol targeting chlorhexidine bathing for patients with medical devices and nasal mupirocin among those who also had a history of MRSA was the cost-effective strategy across a wide range of scenarios from both hospital and payer perspectives.

The outcome evaluated, HOB, is highly relevant as an imminent hospital quality standard, expected to ultimately replace central-line–associated bloodstream infection. HOB is estimated to have a 5-fold higher hospital event rate than central-line–associated bloodstream infection (up to 17-fold in ICUs), involving substantially greater costs and hospital length of stay than was conservatively applied in our analysis.^3,37,38,39,40,41^ For example, others have estimated that each central-line–associated bloodstream infection event can cost anywhere in the range of $18 000 to $95 000.^42,43,44,45^ The greater the incurred cost and length of stay, the more effective decolonization becomes for both averting HOB and reducing cost.

There were several conditions under which UD became the cost-effective strategy in place of TD. The first is when implementation of TD failed to reach patients with medical devices with high success. In the ABATE Infection trial, chlorhexidine was universally provided for daily bathing and showering with resultant adherence for daily bathing of 79%. The adherence is likely even less when chlorhexidine is not automatically present in the room or shower and must be requested as a special order. Second, when the patient case mix involved a high proportion of patients with medical devices or when patients without medical devices had a high risk for HOB, then UD became the cost-effective strategy. This may be relevant to tertiary or quaternary academic and nonacademic medical centers, possibly even selected units, such as step-down units.

There are additional conditions that could favor UD that were not accounted for in this study, such as when a universal approach leads to indirect benefits, potentially by lowering the overall bioburden in a unit. For example, chlorhexidine bathing has been reported to reduce the bacterial bioburden on the hands of health care personnel as well as environmental contamination by reducing human shedding of bacteria^46,47^; indeed, this may be one explanation for the success of UD over TD in the REDUCE MRSA ICU Trial.^2^ Universal decolonization could be favored in a greater number of scenarios if decolonization reduced other infections beyond HOB. For example, decolonization has been reported to reduce surgical site infections, skin and soft tissue infections, bacteriuria, and a wide variety of infections due to multidrug-resistant organisms,^7,8,9,48,49^ including infection-related readmissions.

### Limitations

There are several limitations to this study. First, there is no accepted threshold for what payers or hospitals would be willing to pay to prevent HOB infections, unlike, for example, with dollars per quality-adjusted life year.^20^ In our analysis, relative assessments were used to estimate the ideal strategy, and willingness-to-pay would have to be much higher than our adopted values to favor UD in the base case.

Second, the patient characteristics and their HOB risks reflected the ABATE Infection trial; we believe this population is broadly representative of US community hospitals but may have more limited generalizability to academic medical centers. Third, our models applied a uniform hazard reduction for anyone with a medical device. In reality, comorbidities and other characteristics may change the effectiveness of decolonization. Nevertheless, a wide range of possible outcomes was explored in sensitivity analyses. Fourth, we were conservative in modeling health outcomes. We did not model any outcomes other than HOB and we did not include any long-term outcomes of increased longevity or improved quality of life from avoiding HOB, nor did we capture the health and cost effects resulting from changes to readmissions.

Fifth, we did not account for economies of scale in product costs. Sixth, while staff time would not be expected to differ between bathing with chlorhexidine as opposed to soap, we did not account for the cost between strategies of the additional staff time required to apply mupirocin. Seventh, we did not model any adverse effects from decolonization, but given the rare event rate and the mild nature of reported adverse events in large-scale trials, these effects would be unlikely to change our conclusions. Eighth, we did not model the potential consequences from resistance to decolonization products. However, we did report the number of individuals receiving mupirocin per 1000 patients under each strategy, which was considerably higher in the UD strategy, and may help to inform stewardship discussions pertaining to the use of mupirocin. Ninth, the net costs accruing to the hospital were uncertain and are likely heterogeneous between settings. In some part this would depend on whether the hospital is one of nearly 800 nationwide to receive the Centers for Medicare & Medicaid Services value-based penalty for poor performance on hospital-acquired conditions.^50,51^ However, by way of sensitivity analyses on this and other parameters, we hope to have addressed the specific decision-making contexts of each hospital.

## Conclusions

This decision analytic model on the cost-effectiveness of decolonization vs SOC for HOB in general medical and surgical hospital units found that decolonization reduced costs and provided health benefits compared with the SOC. The findings suggest that, overall, TD was the preferred strategy, but several conditions favored UD.

## Notes

Supplement 1. **eTable 1.** Details of the Modeled Strategies **eTable 2.** Model Input Parameters **eTable 3.** Cost Results by Upstream and Downstream Categories **eFigure.** Impact of Four Key Parameters on Optimal Bathing Strategy, Hospital Perspective

Supplement 2.
Data Sharing Statement
