# Supplementary material for: Universal vs Targeted Chlorhexidine Bathing and Nasal Decolonization in Hospitalized Patients
Source: JAMA Netw Open. 2025 Mar 10;8(3):e250341. doi: 10.1001/jamanetworkopen.2025.0341 (PMC11894500; doi:10.1001/jamanetworkopen.2025.0341)
Supplement: Supplement 1. — eTable 1. Details of the Modeled Strategies eTable 2. Model Input Parameters eTable 3. Cost Results by Upstream and Downstream Categories eFigure. Impact of Four Key Parameters on Optimal Bathing Strategy, Hospital Perspective [file jamanetwopen-e250341-s001.pdf]

Supplemental Online Content

James LP, Stout NK, Avery TR, et al. Universal vs targeted chlorhexidine bathing and nasal decolonization in hospitalized patients. *JAMA Netw Open*. 2025;8(3):e250341. doi:10.1001/jamanetworkopen.2025.034

eTable 1. Details of the Modeled Strategies
eTable 2. Model Input Parameters
eTable 3. Cost Results by Upstream and Downstream Categories
eFigure. Impact of Four Key Parameters on Optimal Bathing Strategy, Hospital Perspective

This supplemental material has been provided by the authors to give readers additional information about their work.

© 2025 James LP et al. *JAMA Network Open*.

eTable 1. Details of the modeled strategies

Strategy	MRSA status	Surgical patient	Medical device	Type of bathing	Mupirocin Use
Standard of Care	MRSA History	Surgical	Device	Chlorhexidine (some)*	No
			No device	Chlorhexidine (some)*	No
		Non-surgical	Device	Routine Soap	No
			No device	Routine Soap	No
	No MRSA History	Surgical	Device	Chlorhexidine (some)*	No
			No device	Chlorhexidine (some)*	No
		Non-surgical	Device	Routine	No
			No device	Routine	No
Universal Decolonization	MRSA History	Surgical	Device	Chlorhexidine	Yes
			No device	Chlorhexidine	Yes
		Non-surgical	Device	Chlorhexidine	Yes
			No device	Chlorhexidine	Yes
	No MRSA History	Surgical	Device	Chlorhexidine	No
			No device	Chlorhexidine	No
		Non-surgical	Device	Chlorhexidine	No
			No device	Chlorhexidine	No
Targeted Decolonization for	MRSA History	Surgical	Device	Chlorhexidine	Yes
			No device	Chlorhexidine	No

© 2025 James LP et al. *JAMA Network Open*.

those with devices				(some)*	
		Non-surgical	Device	Chlorhexidine	Yes
			No device	Routine soap	No
	No MRSA History	Surgical	Device	Chlorhexidine	No
			No device	Chlorhexidine (some)*	No
		Non-surgical	Device	Chlorhexidine	No
			No device	Routine	No

MRSA – methicillin-resistant *S. aureus*

*For surgical patients not receiving the intervention, we assumed 50% would still receive chlorhexidine bathing as standard practice for specific procedures.

© 2025 James LP et al. *JAMA Network Open*.

eTable 2. Model input parameters

Variable Description	Point estimate	Distribution for Probabilistic Sensitivity Analysis	Source	Notes
Health effects				
Baseline probability of HOB during admission:<br><br>among those with: devices, MRSA history, and a surgical admission	0.10976	Beta (alpha = 18, beta = 146)	Personal communication: secondary analysis of trial data	The point estimate is the mean of the two baseline period probabilities. The numerator is the number of events, the denominator is the number of admissions.<br><br>Number of events and non-events are the total in patients in this group, summed across the baseline period in both arms.<br><br>The distribution parameters reflect the total numbers of events (alpha) and non-events (beta). This is converted to a rate (per admission) in order to apply the below HRR. We apply the HRRs from the study, assuming the same ratios apply to the per admission rate as the per inpatient day rate.
Baseline probability of HOB during admission among those with: devices, MRSA history, and a non-surgical admission	0.08075	Beta (alpha = 73, beta = 831)	Personal communication: secondary analysis of trial data	As above
Baseline probability of HOB during admission among those with: devices, no MRSA history, and a surgical admission	0.01458	Beta (alpha = 100, beta = 6759)	Personal communication: secondary analysis of trial data	As above

© 2025 James LP et al. *JAMA Network Open*.

Baseline probability of HOB during admission among those with: devices, no MRSA history, and a non-surgical admission	0.02298	Beta(alpha = 338, beta = 14371)	Personal communication: secondary analysis of trial data	As above
Baseline probability of HOB during admission among those with: no devices, MRSA history, and a surgical admission	0.02283	Beta(alpha = 5, beta = 214)	Personal communication: secondary analysis of trial data	As above
Baseline probability of HOB during admission among those with: no devices, MRSA history, and a non-surgical admission	0.01908	Beta(alpha = 47, beta = 2416)	Personal communication: secondary analysis of trial data	As above
Baseline probability of HOB during admission among those with: no devices, no MRSA history, and a surgical admission	0.00157	Beta(alpha = 54, beta = 34283)	Personal communication: secondary analysis of trial data	As above
Baseline probability of HOB during admission among those with: no devices, no MRSA history, and a non-surgical admission	0.00199	Beta(alpha = 258, beta = 129168)	Personal communication: secondary analysis of trial data	As above
Hazard rate ratio for HOB for patients with devices – treated	0.86	Log-Normal, informed by the reported mean and 95% CI (0.77-0.96). Resulting mean	Personal communication: secondary analysis of trial data	Lognormal chosen as this is a positive variable with positively skewed CIs (as the estimate was obtained from a Cox-PH, i.e. with log link).

© 2025 James LP et al. *JAMA Network Open*.

		(SD) of the log: -0.153 (0.0591).		
Hazard rate ratio for HOB for patients with devices – untreated	1.14	Log-Normal informed by the mean and 95% CI (1.00-1.30). Resulting mean (SD) of the log: 0.129 (0.066).	Personal communication: secondary analysis of trial data	As above
Hazard rate ratio for HOB for patients without devices – treated	1.00	N/A	Assumption	Based on finding of no significant difference in the ABATE Infection trial.[4]
Hazard rate ratio for HOB for patients without devices – untreated	1.00	N/A	Assumption	Based on finding of no significant difference in the ABATE Infection trial.[4]
Probability of having a medical device	0.125	N/A – varied only in deterministic sensitivity analysis	Personal communication: secondary analysis of trial data	
Probability of having a history of MRSA	0.08	N/A – varied only in deterministic sensitivity analysis	Livorsi et al. 2021[45]	This is the median estimate for VHA hospitals. Note that this is higher than the proportion of patients with a documented history of MRSA in the ABATE trial (0.0175).
Probability of having surgery	0.21650	N/A – varied only in deterministic sensitivity analysis	Huang SS, Septimus E, Kleinman K, et al. Lancet 2019[4]	Table 1 in citation. Calculated as the mean of the two proportions of patients having surgery in the intervention period (i.e. the mean across the routine care and decolonization arms).

© 2025 James LP et al. *JAMA Network Open*.

Proportion of surgical patients without a history of MRSA in the standard of care who receive universal bathing with chlorhexidine	0.5	N/A – varied only in deterministic sensitivity analysis	Assumption	We assumed that all patients with a history of MRSA receive universal bathing with chlorhexidine, and nasal mupirocin (See Table S1).
Proportion of patients in the targeted intervention strategy who receive the intervention, *relative* to UD	0.9	N/A – varied only in deterministic sensitivity analysis	Assumption	Varied in sensitivity analysis. When this variable = 1, adherence is the same under TD as in UD.
Costs				
Excess cost of treatment of HOB in 2006 USD	$19,643	Normal with 95% CI (9,026, 30,260)	Kilgore M and Brossette S. Clin Infect Dis 2008.[14]	This estimate was chosen as there was a distribution surrounding it, which we used for the probabilistic sensitivity analysis. It is in line with the base case used in REDUCE MRSA, which was itself calculated from published estimates.<br><br>Values are drawn from this distribution and then inflated using the Consumer Price Index (medical) to 2022 USD.
Consumer Price Index	Various	N/A	Consumer Price Index (medical)[46]	Applied to convert all costs into 2022 USD.
Cost of chlorhexidine bedbath, per day (2012 USD)	$5.52	Gamma(mean = 5.52, s.d. = 1.84)	Petlin A, Schallom M, Prentice D, et al. Crit Care Nursing 2014.[47]	Using prepackaged CHG wipes, assumes one bedbath per day.
Cost of chlorhexidine shower, per day (2014 USD)	$0.875	Gamma(mean = 0.875, s.d. = 0.292)	Petlin A, Schallom M, Prentice D, et al. Crit Care Nursing 2014.[47]	Assumes one bottle of 4% chlorhexidine lasts 2 days.

© 2025 James LP et al. *JAMA Network Open*.

Cost of standard bedbath using soap, per day (2004 USD)	$2.11	Gamma(mean = 2.11, s.d. = 0.70)	Larson EL, Ciliberti T, Chantler C, et al. Am J Crit Care 2004.[48]	Assumes one bedbath per day.
Cost of standard shower using soap, per day (2022 USD)	$0.44	Gamma(mean = 0.44, s.d. = 0.147)	Larson EL, Ciliberti T, Chantler C, et al. Am J Crit Care 2004.[48]<br>Petlin A, Schallom M, Prentice D, et al. Crit Care Nursing 2014.[47]	Obtained cost of soap and basin from Larson et al (0.50 in 2004 USD), and the cost of the basin alone from Petlin et al. (0.35 in 2014 USD). Updated both to 2022 USD and took the difference to arrive at soap only.
Proportion of inpatients having bedbaths	78%	N/A	Huang SS, Septimus E, Kleinman K, et al. Lancet 2019[4]	The remainder (22%) take showers.
Cost of twice daily intranasal mupirocin for 5 days (2005 USD)	$6.23	Gamma (mean = 6.23, s.d. = 2.08)	Courville XF, Tomek IM, Kirkland KB, et al. Infect Contr Hosp Epidemiol 2012[49]<br><br>Young LS, Winston LG. Infect Control Hosp Epidemiol 2006[50]	
Hospital length of stay	6.5 days	IQR 4-8 days	Huang SS, Septimus E, Kleinman K, et al. Lancet 2019[4]	Table 1 in citation. This is used in combination with the cost of bathing materials to calculate the costs of the bathing approach under each strategy.<br><br>In order to construct a probability distribution that adequately matches this interquartile range, we adopted a lognormal distribution with median 5, and chose a mean 6.5 [mean of the log = ln(5); s.d. of the log = sqrt(ln((6.5)/(5))*2)].This provides a 75th centile that almost exactly matches that in the study (i.e., 8). The resulting 25th centile was a little lower than the study at $^{3.}$1, however.

CHG – chlorhexidine gluconate; HOB – hospital-onset bacteremia and fungemia; IQR – interquartile range; MRSA – methicillin-resistant *S. aureus*; SD – standard deviation

© 2025 James LP et al. *JAMA Network Open*.

eTable 3. Cost Results by Upstream and Downstream Categories

	**Payor Perspective Cost per 1,000 Admissions, $**			**Hospital Perspective Cost per 1,000 Admissions, $**	
**Strategy**	**Upstream** (i.e., resulting from bathing and mupirocin administration)	**Downstream** (i.e., resulting from HOB events)		**Upstream** (i.e., resulting from bathing and mupirocin administration)	**Downstream** (i.e., resulting from HOB events)
Targeted Decolonization	23,151 (3,895, 77,796)	186,868 (84,057, 290,007)		23,151 (3,895, 77,796)	146,466 (134,007, 160,423)
Universal Decolonization	35,642 (6,326, 121,241)	184,238 (82,822, 286,019)		35,642 (6,326, 121,241)	144,405 (132,093, 158,331)
Standard of Care	21,568 (3,467, 73,131)	210,532 (94,946, 326,529)		21,568 (3,467, 73,131)	165,015 (151,671, 179,853)

HOB – hospital-onset bacteremia and fungemia

Point estimates shown with 95% uncertainty intervals (UIs) in parentheses.

Upstream costs (i.e., those resulting from bathing and mupirocin administration) were modeled in the same way under payor and hospital perspectives. Downstream costs (i.e., those resulting from HOB events) were informed by a published analysis for the payor perspective. For the hospital perspective, we assumed that the cost accruing to the hospital for each HOB event was $25,000, which included all non-reimbursed costs of providing care, as well as any financial penalties incurred. All costs were recorded as 2022 US dollars, and were inflated from older published estimates where necessary using the Consumer Price Index for medical care.

© 2025 James LP et al. *JAMA Network Open*.

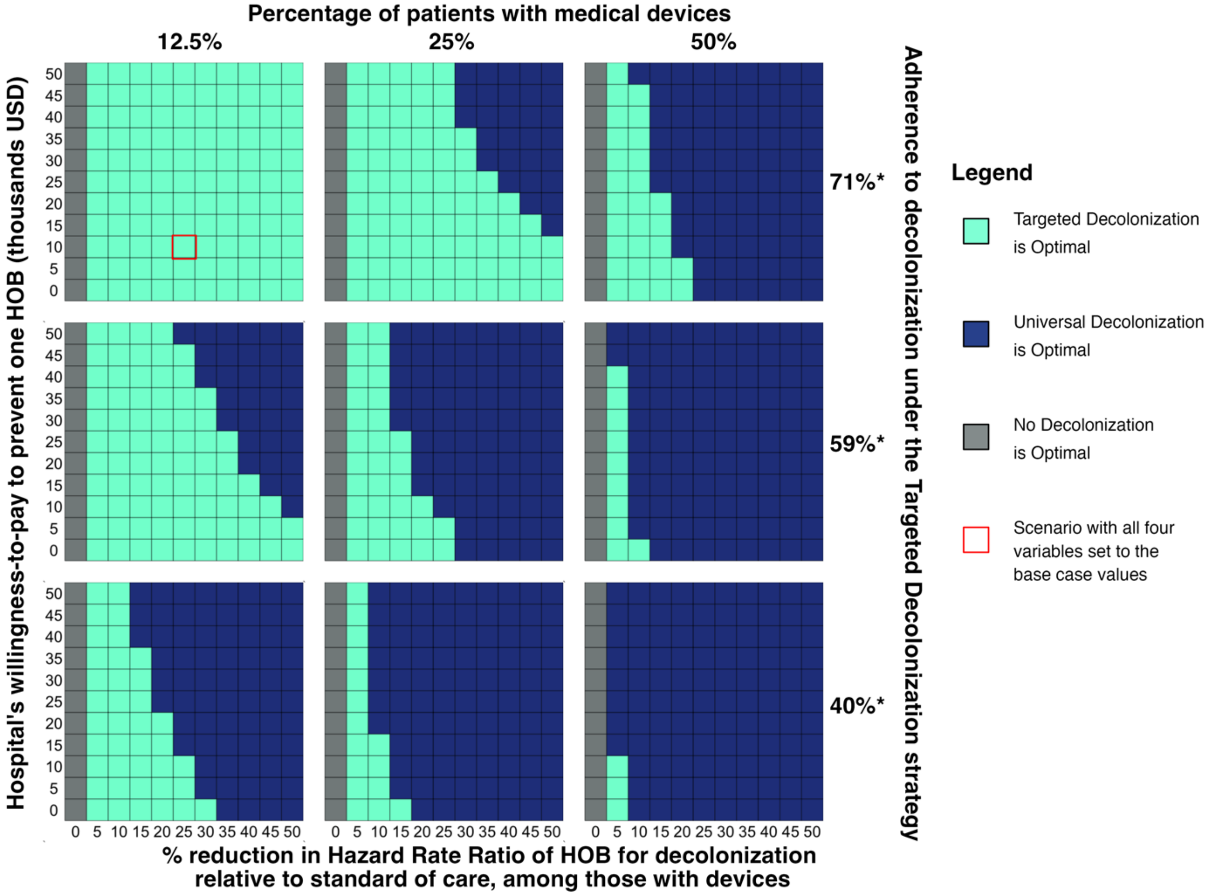

eFigure: Impact of four key parameters on optimal bathing strategy, hospital perspective. The optimal strategy (i.e., that which provided the best value for money) is shown for hospital decision makers, as a function of the proportion of patients with devices, adherence to decolonization under the Targeted Decolonization strategy, the treatment effect for decolonization among those with devices, and the hospital decisionmaker's willingness-to-pay to avoid one HOB. All other variables are held at base case values. *Adherence values of {40%, 59%, 71%} refer to absolute adherence, and equate to {50%, 75%, 90%} *relative* to 79% adherence to chlorhexidine bathing under Universal Decolonization experienced in the ABATE Infection Trial.

HOB – hospital-onset bacteremia and fungemia; USD – United States dollars

© 2025 James LP et al. *JAMA Network Open*.
