# Supplementary material for: Universal vs Targeted Chlorhexidine Bathing and Nasal Decolonization in Hospitalized Patients
Source: JAMA Netw Open. 2025 Mar 10;8(3):e250341. doi: 10.1001/jamanetworkopen.2025.0341 (PMC11894500; doi:10.1001/jamanetworkopen.2025.0341)
Supplement: Supplement 2. — Data Sharing Statement [file jamanetwopen-e250341-s002.pdf]

Data Sharing Statement

James. Universal vs Targeted Chlorhexidine Bathing and Nasal Decolonization in Hospitalized Patients. *JAMA Netw Open*. Published March 10, 2025.
doi:10.1001/jamanetworkopen.2025.0341

Data
Data available: Yes
Data types: Other (please specify)
Additional Information: Model parameters were taken from published literature. For data sharing related to the ABATE Infection Trial, please see data sharing statement from the trial publication (Lancet. 2019. Mar 23;393(10177):1205-1215. PMID: 30850112)
How to access data: All model code is available at https://github.com/lyndonpjames/CEA_CHGBathing [github.com]
When available: With publication

Supporting Documents
Document types: Statistical/analytic code
How to access documents: https://github.com/lyndonpjames/CEA_CHGBathing [github.com]
When available: With publication

Additional Information
Who can access the data: Publicly available on GitHub
Types of analyses: Full code is available for others to use.
Mechanisms of data availability: Freely available
